# Paired Analysis of D-Dimer and Its Correlated Hemostatic Parameters in 30 Dogs with Neoplasms after Tumorectomy

**DOI:** 10.3390/ani13060969

**Published:** 2023-03-07

**Authors:** Chiao-Hsu Ke, Cheng-Chi Liu, Shang-Lin Wang, Chen-Si Lin

**Affiliations:** 1 Department of Veterinary Medicine, School of Veterinary Medicine, National Taiwan University, Taipei 10617, Taiwan; 2 Graduate Institute of Veterinary Clinical Science, School of Veterinary Medicine, National Taiwan University, Taipei 10617, Taiwan; 3 Animal Cancer Center, College of Bioresources and Agriculture, National Taiwan University, Taipei 10617, Taiwan; 4 National Taiwan University Veterinary Hospital, College of Bioresources and Agriculture, National Taiwan University, Taipei 10672, Taiwan

**Keywords:** D-dimer, tumor biomarker, hemostatic parameter, liquid biopsy

## Abstract

**Simple Summary:**

Cancer biomarkers often reflect tumor burden in canine patients; however, these substances with high clinical utility are rarely reported. Hemostatic dysfunction is usually found in patients during tumorigenesis, especially hyper-coagulability. Therefore, aberrant hemostatic parameters can serve as potential indicators for dogs with tumors. Among these assessments, D-dimer has been proposed as a tumor biomarker in human and veterinary oncology. However, research describing the clinical utility and reporting the paired analysis of D-dimer values in the same dogs still needs to be elucidated in clinical veterinary medicine. In the current study, we demonstrated that D-dimer values significantly increased in the dogs with tumors, and the values significantly decreased with the removal of primary tumors. Furthermore, this research also compared other standard hemostatic parameters and revealed that the D-dimer and several hemostatic values are correlated to tumor burden. With the urgent demand to discover valuable biomarkers in canine tumors, our findings showed that several hemostatic parameters could be applicable choices via evidence-based medicine.

**Abstract:**

Previous studies have reported that dogs with neoplasms had elevated D-dimer levels. However, few studies have addressed whether D-dimer could be an indicator of tumor burden. The clinical significance of paired analysis of pre- and post-operation of D-dimer levels in dogs has rarely been described. The present study investigated the values of D-dimer levels and their correlated hemostatic alterations in dogs with surgically removable benign and malignant tumors. This study analyzed 30 clinically healthy and 30 tumor-bearing dogs and evaluated the hemostatic functions including D-dimer, thromboelastography G (TEG G), fibrinogen, activated partial thromboplastin time (aPTT), prothrombin time, and platelet count. The median level of pre-treatment D-dimer was 0.8 µg/mL (range: 0.1–6.3 µg/mL), whereas the control dogs exhibited a median value of 0.1 µg/mL (range: 0.1–0.1 µg/mL, *p* < 0.0001). After tumorectomy, the median levels of D-dimer (*p* < 0.0001), fibrinogen (*p* < 0.0001), TEG G value (*p* < 0.01), and aPTT (*p* < 0.05) were significantly lower than those of the pre-treatment samples. However, further studies are needed to clarify the values of other hemostatic evaluations. The study revealed the clinical significance of D-dimer and its correlated hemostatic parameters by paired analysis in dogs with tumors. Though more cases are needed for solid confirmation, these values could be potential tumor biomarkers for dogs.

## 1. Introduction

Cancer biomarkers have become effective indicators for tumor categorization, malignancy, and prognosis [1]. These substances are produced by tumors and might exist in patients’ body fluids, and they usually reflect the progression of tumors in patients with cancers [2,3]. Therefore, research on cancer biomarkers that can be determined in body fluids, especially in the blood, has gained more attention in both human and veterinary medicine [4]. However, the low estimated rate of successful clinical translation of biomarkers [5] implies that cancer biomarkers with high clinical utility are rarely reported in clinical medicine. Recently, more and more clinical studies have suggested an association between cancer and hemostasis [6,7], and hemostatic dysfunction is usually found in human cancer patients with clinical signs of pulmonary embolism (PE) and disseminated intravascular coagulation (DIC) [8].

Coagulation and fibrinolysis are activated in tumors that promote tumor growth and metastasis by regulating angiogenesis [9]. The pathogenesis of blood coagulation activation in cancers is multifactorial. However, the tumor cells favor the clotting cascade with the appearance of hyper-coagulable states in patients and thus contribute to tumor progression [10]. Moreover, the stimulation of tumor-associated inflammatory cells can activate coagulation and fibrinolysis, which can trigger tumor growth, metastasis, and angiogenesis [11,12]. In response to hyper-coagulability, D-dimer has been widely used in human medicine [13,14,15] because this stable end-product of fibrin degradation is a highly sensitive indicator of activated coagulation and fibrinolysis [16]. Elevated levels of D-dimer have been reported in patients with cancers such as colorectal [17], liver [18], lung [19], and gastric cancers [20]. Likewise, in veterinary medicine, the relationship between tumors and D-dimer has also gained more attention in recent years. Elevated levels of D-dimer were identified in dogs with tumors as compared with those without cancers [21]. Moreover, plasma D-dimer levels are positively associated with cancer malignancy [7,8,22].

Previous studies have reported the clinical utility of D-dimer in veterinary medicine. D-dimer is significantly elevated in dogs with tumors, with high values in dogs suffering from lymphoma and carcinoma [23]. Dogs with malignant tumors [21,23] and distant metastasis [8,23] exhibited elevated D-dimer levels. Boyé et al. [22] demonstrated that plasma D-dimer level could serve as a predictor of prognosis in dogs with lymphoma. Moreover, immunostaining revealed that D-dimer was deposited in both extravascular and intravascular spaces in dogs with gliomas, while no D-dimer immunolabelling was detected in healthy dogs [24]. These findings indicated that D-dimer is an indicator for dogs suffering from different types of tumors. However, the accuracy of this marker remains controversial, and it is unknown whether D-dimer has the potential to serve as a biomarker in the same dogs with medical intervention. Furthermore, to the authors’ best knowledge, no studies in the literature describe the clinical significance of paired analysis of pre- and post-tumorectomy of D-dimer and its correlated hemostatic levels in dogs. Therefore, this study investigated the plasma D-dimer values in control dogs and dogs with tumors. The second purpose was to evaluate several hemostatic parameters before and after tumorectomy in the same dog with neoplasms. This study proposes several values as tumor biomarker candidates in veterinary medicine.

## 2. Materials and Methods

### 2.1. Patient Selection

A total of 30 client-owned dogs with different types of tumors that presented at the National Taiwan University Veterinary Hospital, Taipei, Taiwan, from 2021 to 2022 were recruited (defined as “patients”). Patients of any age, sex, or breed were eligible for enrollment in this retrospective study; however, patients with concurrent diseases unrelated to the tumor diagnosis that could have impacts on the results were excluded. Physical examination, evaluation of superficial draining of lymph nodes, complete biochemistry profile, coagulation and fibrinolysis parameters, and thromboelastography G (TEG G) were evaluated in the patients. Patients previously receiving non-steroidal anti-inflammatory drugs (NSAIDs) or steroids for the last 14 days before blood sampling were also excluded. No patients had received chemotherapy, anti-coagulant drugs, or blood transfusion prior to enrollment. The primary tumors of the dogs were surgically removed. Histopathological diagnosis was employed for all dogs with tumors. Based on the histopathological evaluation, the patients were further distributed into three groups in accordance with tumor origin [25], namely, mesenchymal-type tumors, epithelial-type tumors, and melanoma (other). Control samples (defined as “controls”) from active patients without neoplastic purpose at the National Taiwan University Veterinary Hospital were collected with owner consent. The study was approved by the Institutional Animal Care and Use Committee of National Taiwan University, Taipei, Taiwan (protocol code: IACUC No. NTU110-EL-00095).

### 2.2. Blood Sampling and Hematology

Blood collection from each dog with tumors was performed at two time points, just before and 3 weeks after the surgery. Whole blood with a maximum volume of 2% of the body weight was collected from the cephalic or jugular veins. Blood samples were immediately anticoagulated with 3.2% sodium citrate in a 1:9 ratio. For the TEG test, the whole blood was stored at room temperature for 30 min after sampling until the measurement. For D-dimer, fibrinogen, activated partial thromboplastin time (aPTT), and prothrombin time (PT) calculations, the blood was packed in anti-coagulation tubes and immediately centrifuged at 4000× *g* for 120 s. An automated hematology analyzer (Procyte DX hematology analyzer; IDEXX, Westbrook, ME, USA) was employed to analyze the EDTA-anticoagulated whole blood, including complete blood count (CBC) and platelet count (PLT).

### 2.3. Coagulation Parameters Measurement

Commercial reagents in a validated setup using an automated coagulometer (ACLT op 500, Instrumentation Laboratory, Tokyo, Japan) for aPTT and PT (Syemex CA-500); fibrinogen (RecombiPlasTin 2G, Instrumentation Laboratory) tests were performed as previously described [26]. Normal values for these assays were based on a previous study [8]. The canine plasma samples were tested by the Vcheck D-dimer diagnostic assay (Bionote Ltd., Hwaseong-si, Republic of Korea). D-dimer concentrations were performed on a Bionote Vcheck V2400 analyzer (Bionote Ltd., Hwaseong-si, Republic of Korea).

### 2.4. Thromboelastography

The TEG analysis was performed as previously described [27]. Briefly, the citrated whole-blood samples were activated with a solution of recombinant human tissue factor (TF) at a final concentration of 1:50,000. Before analysis, 20 µL 280 mM CaCl_2_ was added to the reagent cup to re-calcify the samples. Then a 360 µL sample, which was composed of 20 µL prediluted TF, 320 µL citrated whole blood, and the 20 µL re-calcified premix solution, was submitted for 120-min analysis. The TEG parameters were evaluated for all individuals with tumors as previously described [21,28]. G was calculated from MA with G = 5000 × MA/(100 − MA). TEG G is a method to evaluate whole-blood coagulability as normo-, hyper-, or hypo-coagulant. Patients with the TEG G values within the normal range of 3.2–7.2 × 10^3^ dyn/cm^2^ were considered normo-coagulable. Those with TEG G values below 3.2 × 10^3^ dyn/cm^2^ were classified as hypo-coagulable, and those with values above 7.2 × 10^3^ dyn/cm^2^, as hyper-coagulable [8,21].

### 2.5. Statistical Analysis

Data are presented as median and interquartile range (IQR, range from the 25th to the 75th percentile). Normal distribution was examined with D’Agostino and Pearson test and nonparametric tests were utilized for data comparisons. Differences between patients and controls were analyzed using the Mann–Whitney U test. For paired analysis of pre- and post-operative samples from patients, the Wilcoxon matched-pairs signed-rank test was utilized. Receiver operating characteristic (ROC) curves were generated, and the areas under the ROC curves (AUC) were used to determine the adequacy of the D-dimer value as a marker to differentiate controls from patients. According to a previous study [29], the results of AUC could differentiate between non-informative (AUC = 0.5), less accurate (0.5 < AUC ≤ 0.7), moderately accurate (0.7 < AUC ≤ 0.9), highly accurate (0.9 < AUC ≤ 1), and perfect tests (AUC = 1). Statistical analyses were performed in GraphPad Prism Software (GraphPad Prism version 9.0, GraphPad Software, San Diego, CA, USA), and *p* values of <0.05 were considered statistically significant.

## 3. Results

### 3.1. Clinical Characteristics

This study enrolled 30 patients with a median age of 10 years (range: 6–15 years), which was significantly higher than the 30 control individuals, which had a median age of 3.25 years (range: 1.3–6.0 years; *p* < 0.0001). For the patients, the mixed dog was the most common breed encountered (n = 18), with four Siberian Huskies, two Labrador Retrievers, and one each of the following: Golden Retriever, Doberman Pinscher, Miniature Dachshund, Miniature Poodle, Miniature Schnauzer, and Yorkshire Terrier. The gender distribution was 40% males and 60% females in the patient group. For the control group, a total of 30 dogs were recruited, including 12 Formosan dogs, 6 beagles, 4 Border Collies, 4 mixed dogs, 2 Irish Setter dogs, 1 Golden Retriever, and 1 standard Poodle. Sexes were similarly represented (13 male and 17 female dogs). The detailed signalments of the control dogs are summarized in Table S1.

Tumor types of individual dogs were diagnosed by histopathology. Of the 30 dogs with tumors in this study, there were seven cutaneous mast cell tumor (cMCT) cases, seven mammary gland tumor (MGT) patients, four melanoma cases, three anal sac tumor cases, two soft tissue sarcoma cases, two lipoma cases, and one each of osteosarcoma, fibrosarcoma, transmissible venereal tumor, oral squamous cell carcinoma, and thyroid carcinoma. The histopathological tumor types and the detailed characteristics of all patients are summarized in Table S2.

### 3.2. Elevated Plasma D-Dimer Values in Tumor-Bearing Dogs

**Figure 1:**
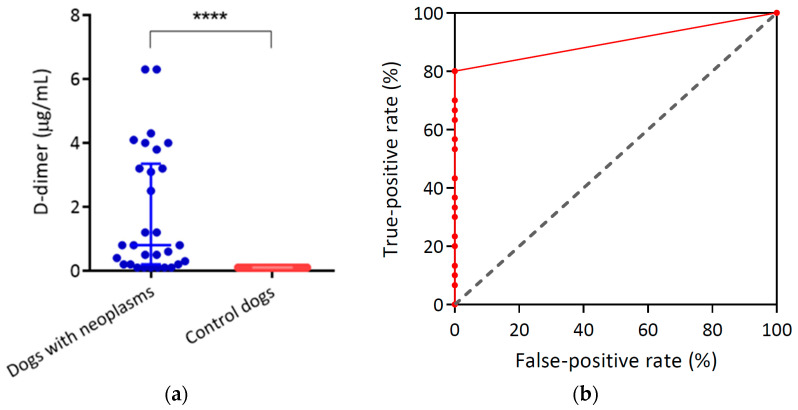
D-dimer concentrations in plasma of all dogs with neoplasms (n = 30) and control dogs (n = 30). (**a**) The median values of D-dimer in cancer and control dogs were 0.8 ± 3.15 and 0.1 ± 0.0 µg/mL, respectively (*p* < 0.0001); (**b**) The ROC curve of detection of D-dimer. The area under the ROC curve (AUC) is 0.90 under a 95% confidence interval of 0.8116–0.9884, with significant difference (*p* < 0.0001). ****, *p* < 0.0001.

Plasma D-dimer levels were analyzed in all recruited dogs. The distribution of D-dimer concentrations measured in the samples of patient dogs (before and after treatments) and control dogs is shown in Table 1. The median D-dimer concentrations in all dogs with tumors and control individuals were 0.8 µg/mL (range: 0.1–6.3 µg/mL) and 0.1 µg/mL, respectively. The findings revealed significantly elevated D-dimer levels in tumor-bearing dogs (Figure 1a, *p* < 0.0001). The ability of D-dimer to distinguish clinical cases from control individuals was assessed by the ROC, and the AUC was evaluated. The AUC was 0.900 with a 95% confidence interval (CI) of 0.8116 to 0.9884, which indicated the highly accurate diagnostic performance of D-dimer to discriminate between patient and control dogs in this population (Figure 1b, *p* < 0.0001). Additional diagnostic test parameters were calculated for D-dimer. The diagnostic sensitivity and specificity of D-dimer varied by the selected cut-off value. As shown in Table 2, at 0.15 µg/mL, the sensitivity was 80% (95% CI: 61.43–92.29%) and the specificity was 100% (95% CI: 88.43–100.0%). These results indicated that D-dimer values have a high potential to be designed as a biomarker to distinguish between patient and control dogs with high diagnostic power.

### 3.3. Decreased D-Dimer Values following Tumorectomy

**Figure 2:**
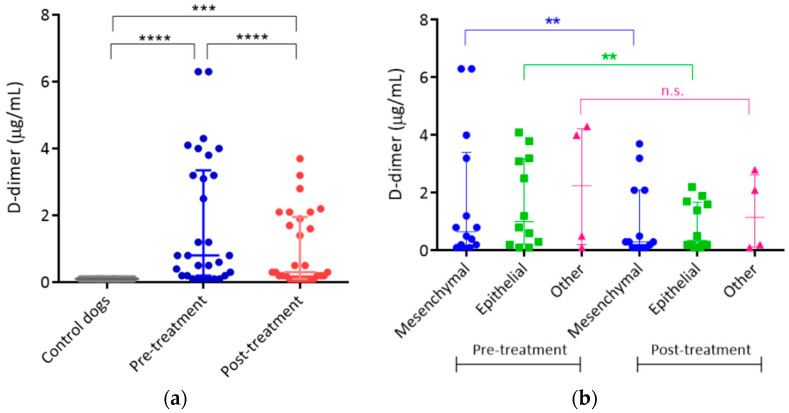
Alteration of D-dimer concentrations in plasma of different types of tumor-bearing dogs before and after treatment. (**a**) The median values of D-dimer are 0.1, 0.8 ± 3.15, and 0.3 ± 1.85 µg/mL in control, pre-treatment, and post-treatment dogs, respectively. The D-dimer values significantly decreased after the operation in all the individuals with tumors (*p* < 0.0001) and the values were significantly elevated in dogs with tumors before (*p* < 0.0001) and after (*p* < 0.001) tumorectomy compared with the control dogs; (**b**) D-dimer significantly decreased in dogs suffering from mesenchymal (n = 14, *p* = 0.0039) and epithelial tumors (n = 12, *p* = 0.0039) after treatment. However, there are no obvious changes in the other dogs with neoplasms (n = 4, *p* = 0.25). Before treatment, the median values of D-dimer were 0.65 ± 3.23, 1.00 ± 2.95, and 2.25 ± 4.03 µg/mL in mesenchymal, epithelial, and melanoma tumor-bearing dogs, respectively. After treatment, the median values of D-dimer were 0.30 ± 2.00, 0.35 ± 1.48, and 1.15 ± 2.50 µg/mL in mesenchymal, epithelial, and melanoma tumor-bearing dogs, respectively. No statistical differences were found among these three populations before or after treatment (*p* > 0.05). *p* values < 0.05 were considered to indicate statistical significance. **, *p* < 0.01; ***, *p* < 0.001; ****, *p* < 0.0001; n.s., no significant difference.

The D-dimer level could be a potential biomarker to differentiate between healthy dogs and dogs with tumors with high sensitivity and specificity (Table 2). To further determine the correlation between D-dimer values and tumor burden, we measured the values at two time points, before and after the operation, respectively, in the same dogs with tumors. Before treatment, the median value of D-dimer was 0.8 µg/mL (range: 0.1–6.3 µg/mL), whereas this parameter obviously decreased to 0.3 µg/mL (range: 0.1–3.7 µg/mL) after tumorectomy (Figure 2a, *p* < 0.0001, dot plots in blue and red). Compared to the control dogs, however, D-dimer levels significantly increased in the dogs with tumors whether or not the tumors were removed (Figure 2a, control vs. pre-treatment: *p* < 0.0001; control vs. post-treatment: *p* < 0.001). Furthermore, to clarify whether the tumor types could result in different D-dimer values, we categorized the 30 dogs with tumors into three major populations, including mesenchymal-type tumors, epithelial-type tumors, and melanomas. As shown in Figure 2b, D-dimer values significantly regressed in mesenchymal (n = 14, *p* < 0.01, dot plots in blue) and epithelial (n = 12, *p* < 0.01, dot plots in green) tumor-bearing dogs after treatment. In contrast, there was no significant difference in melanoma dogs (n = 4, *p* = 0.25, dot plots in pink).

### 3.4. A Significant Decline in Hemostasis-Related Parameters in the Dogs with Tumors following Tumorectomy

**Figure 3:**
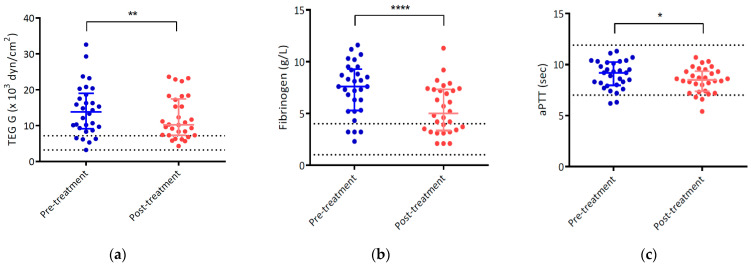

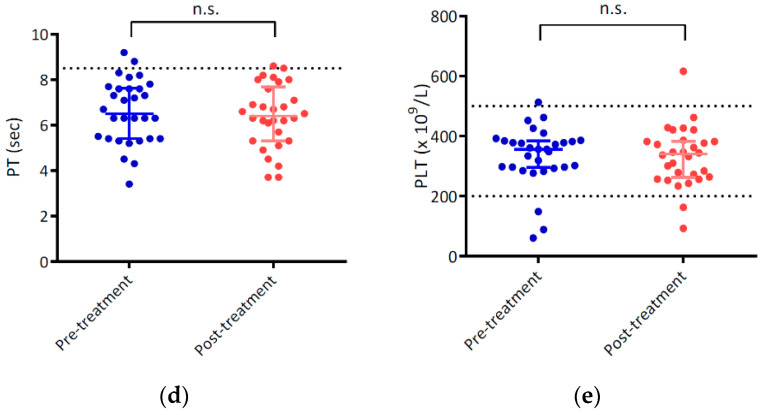
Differences of (**a**) TEG G, (**b**) fibrinogen, (**c**) aPPT, (**d**) PT, and (**e**) PLT before and after tumorectomy. The dotted lines represent the reference interval (TEG G: 3.2–7.2 × 10^3^ dyn/cm^2^; fibrinogen: 1–4 g/L; aPPT: 7–11.9 s; PT: < 8.5 s; PLT: 200–500 10^9^/L). Statistical analysis was performed using GraphPad Prism v9. To determine significant differences between before and after operation, the Wilcoxon matched-pairs signed-rank test was utilized and *p* values < 0.05 were considered as indicating statistical difference. Data are presented as median ± interquartile range. TEG G, thromboelastography G; aPPT, activated partial thromboplastin time; PT, prothrombin time; PLT, platelet count; *, *p* < 0.05; **, *p* < 0.01; ****, *p* < 0.0001; n.s., no significant difference.

Routine coagulation tests, including D-dimer, TEG G, fibrinogen, aPTT, PT, and PLT, were assessed in all patients (Table S2). TEG G is a method to evaluate whole-blood coagulability, and it is usually used to determine hyper-coagulability [8,21]. The distribution according to TEG coagulability of the participants with tumors is described in Table S3. A hyper-coagulable profile was found in 25 of the 30 dogs investigated (83.3%) initially, whereas five dogs (16.7%) were normo-coagulable. Among all the dogs with tumors, in addition to the elevated TEG G levels (median: 13.80 dyn/cm^2^; range: 3.20–32.60, Figure 3a dot plots in blue), D-dimer and fibrinogen were also over-produced in these dogs before tumorectomy; the median values were 0.80 µg/mL (range: 0.10–6.30 µg/mL, Figure 2a dot plots in blue) and 7.60 g/L (range: 2.30–11.60 g/L, Figure 3b dot plots in blue), respectively. Furthermore, the median values of D-dimer, TEG G, and fibrinogen significantly decreased to 0.30 µg/mL (range: 0.10–3.70 µg/mL, *p* < 0.0001, Figure 2a dot plots in red), 10.25 dyn/cm^2^ (range: 4.30–23.60 dyn/cm^2^, *p* < 0.01, Figure 3a dot plots in red), and 5.00 g/L (range: 2.10–11.30 g/L, *p* < 0.0001, Figure 3b dot plots in red) after tumorectomy. In contrast, the levels of aPTT, PT, and PLT remained normal before and after the treatments. The levels of aPTT significantly (*p* < 0.05) decreased to 8.5 s (range: 5.40–10.70 s, Figure 3c dot plots in red) from 9.20 s (range: 6.20–11.30 s, Figure 3c dot plots in blue) after the surgery. However, the median PT values were 6.50 s (range: 3.40–9.20 s, Figure 3d dot plots in blue) and 6.40 s (range: 3.70–8.60 s, Figure 3d dot plots in red) before and after treatment, without a significant difference (*p* = 0.4711). After the treatments, the levels of PLT remained stable, with median levels of 356.0 × 10^9^/L (range: 61.0–513.0 × 10^9^/L, Figure 3e dot plots in blue) and 340.5 × 10^9^/L (range: 93.0–616.0 × 10^9^/L, Figure 3e dot plots in red) before and after treatment (*p* = 0.5125), respectively.

## 4. Discussion

This study provides evidence that hyper-coagulability-related parameters could be used as desirable indicators in dogs after operative treatments. In the current study, D-dimer, TEG G, fibrinogen, and aPTT levels were significantly depressed after tumorectomy. This study also demonstrated that D-dimer levels were substantially higher in the tumor-bearing dogs than in the controls; levels of these biomarkers then decreased after treatment, suggesting their potential as indicators of tumor burden. However, several limitations, such as the small sample size, the presence of outliers, and the diagnosis heterogenicity of this retrospective study, possibly impact these results. Further studies are highly recommended to verify these findings.

Almost all (83.3%) the investigated individuals in this study exhibited elevated hyper-coagulability-related parameters, which is regarded as the indicator of cancer angiogenesis and is predictive of poor prognosis [30]. This finding is in line with a previous study, which reported that hyper-coagulability is the most common hemostatic state observed in dogs with neoplasia [8]. We speculate that the elevated D-dimer, fibrinogen, and TEG G values may have resulted from the pro-inflammatory changes arising within the tumor microenvironment [31,32]. Therefore, after the surgery, even though these parameters significantly decreased (because of the tumorectomy in situ), the inflammatory status in the post-operative dogs still existed, which caused the parameters to remain at elevated levels. Although the exact mechanisms underlying the association between D-dimer level and cancers remains unclear, the dysregulation of the blood coagulation system is considered to be associated with tumorigenicity [33]. One probable explanation might be that the tissue factor-expressing tumor cells could activate thrombin in the tumor microenvironments and secrete pro-inflammatory factors, thereby activating coagulation–fibrinolysis and plasminogen activators [12,21,34]. In addition to the cancer cells, tumor-associated fibroblasts and macrophages can also trigger neo-angiogenesis and the formation of cross-linked fibrin in the extracellular matrix by secreting several growth factors such as vascular endothelial growth factor and tissue factor [35]. The repeated formation-and-degradation cycles of the fibrin matrix result in increased fibrin degradation products, which are known as D-dimer [35,36,37]. Therefore, the tumors and the tumor-infiltrating immune cells simultaneously contribute to the accumulation of D-dimer. In the current study, therefore, elevated levels of D-dimer were found in the patients with tumors as compared with the control dogs, and the D-dimer levels in the dogs with tumors significantly declined after the tumorectomy, potentially echoing these possible mechanisms. Notably, two dogs in the patient group displayed D-dimer (above 6 µg/mL), and another two tumor-bearing dogs exhibited TEG G values (29.3 and 32.6 dyn/cm^2^) much higher than those of the rest of the group. The authors considered these are individual differences and thus retained these values for comparison. However, the changes in D-dimer levels in melanoma-bearing dogs were not significant. One possible explanation is that the relatively small population (n = 4) failed to represent all melanoma dogs, despite the finding of a declining trend after the operation.

Previous studies have defined an approximate range of 0.1–0.5 µg/mL as reference intervals of D-dimer in normal dogs [23,38], which is highly similar to our findings. The current study revealed that the D-dimer concentration of 30 healthy dogs was 0.1 µg/mL. Several studies have distinguished tumor-bearing and normal individuals using a cut-off value of 0.5 µg/mL [8,22]. The median progression-free survival (PFS) for dogs with lymphoma before treatment was 104 days with a D-dimer level <0.5 µg/mL, compared with 54 days for dogs with a D-dimer level above 0.5 µg/mL (*p* = 0.011). The median overall survival (OS) was 169 days in the dogs with a D-dimer level < 0.5 µg/mL, whereas the survival time significantly decreased to 93 days in dogs with a D-dimer >0.5 µg/mL before the medical intervention (*p* = 0.003) [22]. We further allocated all the dogs with tumors into three groups to evaluate whether the origin of the tumors would have an impact on the D-dimer levels. As shown in Figure 2b, there was no significant difference among the three groups, which is relatively in line with a previous study reporting that D-dimer levels were highly similar among connective-, epithelial-, lymphatic-, and exocrine-type tumors [23]. Thus, we speculated that the origins of the tumors might play a minor role in D-dimer formation.

This study has some limitations. The elevated D-dimer levels in human patients with malignant tumors are highly associated with the increased risk of venous thromboembolism (VTE) [13,39]. No dogs with tumors in the current study were found to display systemic VTE, which is in line with a previous clinical study [22]. However, asymptomatic VTE influencing cancer formation cannot be excluded. In addition, dogs with metastatic tumors harbored higher D-dimer levels than dogs with non-metastatic tumors [23], but the D-dimer levels of the dogs with or without tumors metastasis were not evaluated in the current study. This study only compared the D-dimer levels twice in the same individual, and post-treatment examinations were not routinely performed. Therefore, little information about monitoring tumor progression for an extended period is reported in the study. Last, there is insufficient evidence to clarify precisely how hyper-coagulability should be defined in companion animals. Other TEG variables and/or parameters should be considered and evaluated in future studies. Because of the limited sample size, the authors failed to assess the values of different types of tumors and the malignancy of tumors. Evaluating various types of tumors and benign and malignant tumors as one group may also cause bias in the results. Future investigations with comprehensive blood examinations, which could elucidate other hemostatic diseases, and with a long-term prognosis, will be necessary to assess the diagnostic accuracy of D-dimer in dogs with tumors and the correlation with disease progression.

This study demonstrated that dogs with tumors usually exhibited elevated levels of D-dimer; the authors also found that elevated fibrinogen and D-dimer levels in dogs with tumors significantly decreased after tumorectomy. Elevated fibrinogen has been found in dogs with mammary carcinoma, lymphoma, and sarcoma [8,40], which corroborated our findings. Higher levels of D-dimer and fibrinogen were found in the dogs with distant metastasis compared with those without invasive tumors. However, the values of aPTT, PT, or PLT were not correlated to the disease progression [8]. These results indicated that the alterations of D-dimer and fibrinogen levels potentially are indicators for tumor burden with other traditional hemostatic assays. However, the authors propose that the clinical value of fibrinogen levels still needs to be considered because the accuracy of this marker remains controversial. Fibrinogen levels were not statistically different between dogs bearing benign and malignant tumors, whereas D-dimer values significantly increased in the individuals with malignant tumors compared to the benign groups [21]. Anjos et al. reported that fibrinogen levels were significantly elevated in mast cell tumor-bearing dogs (n = 9), while there were no statistical differences between mammary carcinoma (n = 30), hemangiosarcoma (n = 6), and lymphoma (n = 10) groups compared to the control groups [41]. Similarly, in human studies, Çalışkan et al. [42] and Hong et al. [43] found that there was no difference between fibrinogen levels in human cancer patients but also revealed that plasma D-dimer levels were elevated in these patients. These results were inconsistent with our findings, and further studies are highly recommended to elucidate the underlying mechanism and clinical significance of fibrinogen levels in tumor-bearing dogs.

## 5. Conclusions

In conclusion, to the authors’ best knowledge, this study is the first to report the clinical significance of paired analysis of pre-and post-operation of D-dimer and its correlated hemostatic parameters in the same individuals. The D-dimer concentrations were elevated in the investigated patients with low tumor-specificity and significantly decreased after the operation, revealing that D-dimer can be a potential tumor biomarker. Furthermore, we also found that changes in D-dimer, TEG G, fibrinogen, and aPTT might serve as indicators of tumor burden; however, the accuracy and clinical significance of other variables still need to be further evaluated. To sum up, with the urgent demand in veterinary medicine, this study provides several hemostatic parameters that have the potential to serve as tumor biomarkers, via evidence-based medicine.

## Figures and Tables

**Table 1:** Different D-dimer concentration distributions in control individuals and dogs with specific tumor types before and after treatment.

	D-Dimer Concentration (µg/mL)				
	0.1–0.5	0.5–1	1–2	2–3	> 3
Control dogs (n = 30)	30	0	0	0	0
Dogs with neoplasms, pre-treatment (n = 30)	11	6	2	1	10
Mast cell tumor (n = 7)	3	2	-	-	2
Soft tissue sarcoma (n = 2)	-	-	-	-	2
Lipoma (n = 2)	2	-	-	-	-
Osteosarcoma (n = 1)	-	1	-	-	-
Fibrosarcoma (n = 1)	1	-	-	-	-
Transmissible venereal tumor (n = 1)	-	-	1	-	-
Mammary gland tumor (n = 7)	2	1	1	1	2
Anal sac tumor (n = 3)	1	1	-	-	1
Oral squamous cell carcinoma (n = 1)	1	-	-	-	-
Thyroid carcinoma (n = 1)	-	-	-	-	1
Melanoma (n = 4)	1	1	-	-	2
Dogs with neoplasms, post-treatment (n = 30)	17	2	4	5	2
Mast cell tumor (n = 7)	5	-	-	1	1
Soft tissue sarcoma (n = 2)	-	-	-	1	1
Lipoma (n = 2)	2	-	-	-	-
Osteosarcoma (n = 1)	1	-	-	-	-
Fibrosarcoma (n = 1)	1	-	-	-	-
Transmissible venereal tumor (n = 1)	-	1	-	-	-
Mammary gland tumor (n = 7)	3	1	2	1	-
Anal sac tumor (n = 3)	2	-	1	-	-
Oral squamous cell carcinoma (n = 1)	1	-	-	-	-
Thyroid carcinoma (n = 1)	-	-	1	-	-
Melanoma (n = 4)	2	-	-	2	-

**Table 2:** Sensitivity and specificity of D-dimer.

D-Dimer Cut-Off (µg/mL)	Sensitivity (%)	95% CI	Specificity (%)	95% CI *
>0.15	80.00%	61.43–92.29%	100%	88.43–100.0%
>0.25	70.00%	50.60–85.27%	100%	88.43–100.0%
>0.35	66.67%	47.19–82.71%	100%	88.43–100.0%
>0.45	63.33%	43.86–80.07%	100%	88.43–100.0%
>0.55	56.67%	37.43–74.54%	100%	88.43–100.0%

* CI: confidence interval.

## Data Availability

The data presented in this study are available on request from the corresponding author.

## Supplementary Materials

The following supporting information can be downloaded at: https://www.mdpi.com/article/10.3390/ani13060969/s1, Table S1: Signalment of the control dogs; Table S2: Signalment and the results of the hemostatic parameters before and after tumorectomy in dogs with tumors; Table S3: Distribution of 30 dogs with tumors based on thromboelastography G determined coagulability following tumorectomy.
