# Supplementary material for: Paired Analysis of D-Dimer and Its Correlated Hemostatic Parameters in 30 Dogs with Neoplasms after Tumorectomy
Source: Animals (Basel). 2023 Mar 7;13(6):969. doi: 10.3390/ani13060969 (PMC10044208; doi:10.3390/ani13060969)
Supplement: Supplementary file 1 [file animals-13-00969-s001.zip › animals-2210147-supplementary.pdf]

animals MDPI

# animals-2210147 Supplementary Information

**Table S1.** Signalment of the control dogs.

| | Breed | Reproductive status | Age (years) | Body weight (kg) | D-dimer (μg/mL) |
|---|---|---|---|---|---|
| 1 | Beagle | Male intact | 3.5 | 10 | 0.1 |
| 2 | Beagle | Male castrated | 2.0 | 12 | 0.1 |
| 3 | Beagle | Male castrated | 4.0 | 11 | 0.1 |
| 4 | Beagle | Female spayed | 2.0 | 9 | 0.1 |
| 5 | Beagle | Female spayed | 3.8 | 10 | 0.1 |
| 6 | Beagle | Female spayed | 4.5 | 8 | 0.1 |
| 7 | Border Collie | Male castrated | 2.5 | 22 | 0.1 |
| 8 | Border Collie | Male castrated | 3.0 | 25 | 0.1 |
| 9 | Border Collie | Male castrated | 4.0 | 17 | 0.1 |
| 10 | Border Collie | Female spayed | 3.5 | 19 | 0.1 |
| 11 | Formosan | Male intact | 1.8 | 50 | 0.1 |
| 12 | Formosan | Male intact | 2.6 | 55 | 0.1 |
| 13 | Formosan | Female intact | 1.3 | 55 | 0.1 |
| 14 | Formosan | Female intact | 1.3 | 55 | 0.1 |
| 15 | Formosan | Female intact | 1.5 | 52 | 0.1 |
| 16 | Formosan | Female intact | 3.0 | 45 | 0.1 |
| 17 | Formosan | Female intact | 3.5 | 50 | 0.1 |
| 18 | Formosan | Female intact | 3.5 | 50 | 0.1 |
| 19 | Formosan | Female intact | 4.8 | 50 | 0.1 |
| 20 | Formosan | Female intact | 5.5 | 48 | 0.1 |
| 21 | Formosan | Female spayed | 6.0 | 45 | 0.1 |
| 22 | Formosan | Female spayed | 6.0 | 43 | 0.1 |
| 23 | Golden Retriever | Female spayed | 2.0 | 27 | 0.1 |
| 24 | Irish Setter | Male intact | 2.5 | 28 | 0.1 |
| 25 | Irish Setter | Male castrated | 2.5 | 30 | 0.1 |
| 26 | Mixed | Male castrated | 2.5 | 22 | 0.1 |
| 27 | Mixed | Male castrated | 4.0 | 17 | 0.1 |
| 28 | Mixed | Female spayed | 4.0 | 22 | 0.1 |
| 29 | Mixed | Female spayed | 6.0 | 19 | 0.1 |
| 30 | Standard Poodle | Male castrated | 2.5 | 18 | 0.1 |

**Table S2. Signalment and the results of the hemostatic parameters before and after tumorectomy in dogs with tumors.** TEG G, thromboelastography G; aPPT, activated partial thromboplastin time; PT, prothrombin time; PLT, platelet count; MGT, mammary gland tumor; cMCT, cutaneous mast cell tumor; SCC, squamous cell carcinoma; OSA, osteosarcoma; TVT, transmissible venereal tumor; AST, anal sac tumor; FSA, fibrosarcoma; STS, soft tissue sarcoma. .

| No. | Breed | Reproductive status | Age (years) | Body weight (kg) | Tumor type | Benign/ Malignant | TNM | WHO stage | D-dimer (μg/mL) | | TEG G (dyn/$cm^2$) | | aPTT (sec) | | PT (sec) | | Fibrinogen (g/L) | | PLT ($10^9$) | |
|---|---|---|---|---|---|---|---|---|---|---|---|---|---|---|---|---|---|---|---|---|
| | | | | | | | | | Pre-treatment | Post-treatment | Pre-treatment | Post-treatment | Pre-treatment | Post-treatment | Pre-treatment | Post-treatment | Pre-treatment | Post-treatment | Pre-treatment | Post-treatment |
| 1 | Mixed | Female intact | 10 | 16 | MGT | Malignant | T2N0M0 | II | 2.5 | 1.4 | 6.5 | 7.3 | 8 | 8.1 | 6.3 | 6.2 | 7.6 | 3.2 | 297 | 310 |
| 2 | Mixed | Male intact | 11 | 27.5 | Lipoma | Benign | --- | --- | 0.2 | 0.2 | 3.2 | 4.3 | 9.2 | 8.2 | 5.3 | 4.5 | 8.1 | 3.9 | 277 | 284 |
| 3 | Doberman Pinscher | Male castrated | 14 | 43.6 | cMCT[1] | High | --- | I[2] | 0.8 | 0.3 | 20.4 | 15.3 | 11.3 | 8.9 | 5.2 | 5.7 | 10.3 | 9.2 | 378 | 347 |
| 4 | Mixed | Male intact | 8 | 26 | Melanoma | Malignant | T1N1M0 | III | 4.0 | 2.1 | 10.7 | 9.2 | 9.8 | 5.4 | 4.5 | 5.3 | 11.6 | 8.2 | 293 | 256 |
| 5 | Mixed | Female spayed | 12 | 22.7 | MGT | Benign | --- | --- | 0.8 | 0.5 | 16.3 | 18.4 | 6.2 | 8.4 | 7.6 | 6.5 | 7.3 | 6.9 | 462 | 428 |
| 6 | Mixed | Female intact | 13 | 13 | cMCT | Low | --- | I | 0.2 | 0.3 | 9.7 | 10.3 | 7.9 | 9.1 | 5.4 | 5.1 | 8.8 | 11.3 | 384 | 337 |
| 7 | Mixed | Male intact | 10 | 26.5 | SCC | Malignant | T1N0M0 | I | 0.2 | 0.2 | 23.2 | 22.4 | 10.7 | 9.6 | 3.4 | 3.7 | 9.5 | 7.2 | 376 | 257 |
| 8 | Retrievers (Labrador) | Female spayed | 14 | 41.2 | MGT | Benign | --- | --- | 0.3 | 0.1 | 15.9 | 18.2 | 10.4 | 8.6 | 7.6 | 8 | 10.2 | 7.3 | 392 | 347 |
| 9 | Retrievers (Golden) | Male intact | 9 | 27.3 | cMCT | High | --- | I | 4 | 2.1 | 29.3 | 23.2 | 9.5 | 9.3 | 7.3 | 6.7 | 8.3 | 7.4 | 283 | 279 |
| 10 | Mixed | Male castrated | 11 | 21.8 | OSA | Malignant | T1N0M0 | IA | 0.8 | 0.3 | 13.3 | 10.9 | 10.4 | 10.7 | 8.2 | 6.2 | 10.7 | 7.2 | 356 | 377 |
| 11 | Miniature Poodle | Female intact | 10 | 6.3 | Melanoma | Malignant | T1N0M0 | I | 0.5 | 0.2 | 32.6 | 23.6 | 7.4 | 6.8 | 8.1 | 5.3 | 3.2 | 2.1 | 285 | 273 |
| 12 | Mixed | Female intact | 11 | 23.5 | cMCT | Low | --- | I | 0.4 | 0.1 | 10.3 | 11.7 | 11.1 | 9.5 | 6.3 | 6.9 | 7.6 | 5.2 | 382 | 362 |
| 13 | Mixed | Male castrated | 15 | 38.3 | TVT | Benign | --- | --- | 1.2 | 0.5 | 20.8 | 17.2 | 7.6 | 7.4 | 4.3 | 3.7 | 8.5 | 7.7 | 297 | 301 |
| 14 | Mixed | Female intact | 7 | 18 | MGT | Malignant | T3N1M0 | IV | 3.2 | 1.9 | 8.3 | 18.3 | 7.2 | 7.1 | 7.1 | 4.9 | 6.3 | 4.2 | 348 | 332 |
| 15 | Mixed | Female castrated | 13 | 23.2 | AST | Malignant | T1N0M0 | I | 0.6 | 0.2 | 20.3 | 22.9 | 10.2 | 9.8 | 8.8 | 7.9 | 9.5 | 7.4 | 452 | 421 |
| 16 | Mixed | Female spayed | 10 | 30 | cMCT | Low | --- | I | 0.5 | 0.1 | 17.5 | 9.3 | 8.2 | 7.3 | 5.4 | 6.1 | 6.3 | 3.5 | 61 | 93 |
| 17 | Mixed | Female intact | 8 | 3.7 | MGT | Benign | --- | --- | 0.1 | 0.1 | 10.2 | 6.8 | 8.3 | 8.0 | 7.3 | 6.6 | 9.2 | 6.3 | 410 | 616 |
| 18 | Mixed | Male intact | 8 | 10 | Melanoma | Malignant | T2N0M0 | II | 4.3 | 2.8 | 5.3 | 5.7 | 9.5 | 8.7 | 5.3 | 4.2 | 7.3 | 3.2 | 361 | 382 |
| 19 | Mixed | Female spayed | 7 | 11.9 | Lipoma | Benign | --- | --- | 0.1 | 0.1 | 18.6 | 12.3 | 10.1 | 9.3 | 6.3 | 6.8 | 5.3 | 2.1 | 302 | 253 |
| 20 | Mixed | Female spayed | 10 | 5.52 | FSA | Malignant | T3N1M1 | IV | 0.1 | 0.1 | 16.3 | 10.2 | 9.2 | 8.3 | 7.8 | 8.0 | 2.3 | 2.1 | 89 | 163 |
| 21 | Yorkshire Terrier | Male castrated | 6 | 4.28 | STS | Malignant | T3N1M1 | IV | 6.3 | 3.7 | 6.3 | 5.8 | 8.9 | 8.4 | 6.3 | 6.3 | 11.2 | 7.9 | 356 | 382 |

| | | | | | | | | | | | | | | | | | | | | |
|---|---|---|---|---|---|---|---|---|---|---|---|---|---|---|---|---|---|---|---|---|
| 22 | Retrievers (Labrador) | Female spayed | 11 | 12.5 | Melanoma | Malignant | T1N0M0 | I | 0.1 | 0.1 | 23.7 | 15.3 | 6.3 | 6.6 | 7.7 | 8.1 | 8.7 | 4.6 | 513 | 462 |
| 23 | Siberian Huskies | Female spayed | 14 | 21.6 | cMCT | High | --- | II | 6.3 | 3.2 | 15.3 | 11.2 | 8.3 | 7.2 | 7.2 | 7.6 | 4.3 | 4.8 | 386 | 427 |
| 24 | Siberian Huskies | Male intact | 10 | 23 | AST | Malignant | T2N0M0 | II | 3.1 | 1.6 | 10.1 | 6.3 | 7.7 | 7.2 | 5.4 | 6.2 | 6.8 | 4.2 | 378 | 372 |
| 25 | Miniature Dachshunds | Female intact | 13 | 6.9 | MGT | Malignant | T3N0M0 | III | 3.8 | 2.2 | 14.8 | 7.4 | 8.6 | 8.7 | 6.3 | 7.1 | 8.2 | 5.7 | 149 | 264 |
| 26 | Siberian Huskies | Female spayed | 9 | 26.7 | MGT | Benign | --- | --- | 1.2 | 0.2 | 8.7 | 6.2 | 9.3 | 10.2 | 8.3 | 8.6 | 3.2 | 3.4 | 319 | 386 |
| 27 | Schnauzers | Female spayed | 10 | 21.6 | cMCT | Low | --- | II | 0.1 | 0.1 | 9.3 | 8.4 | 10.3 | 9.8 | 7.6 | 8.2 | 5.2 | 3.7 | 334 | 234 |
| 28 | Siberian Huskies | Male intact | 10 | 23 | STS | Malignant | T3N1M0 | III | 3.2 | 2.1 | 12.1 | 8.3 | 8.5 | 8.6 | 5.5 | 6.3 | 3.2 | 3.1 | 298 | 243 |
| 29 | Mixed | Female spayed | 9 | 19 | Thyroid carcinoma | Malignant | T3N1M0 | III | 4.1 | 1.7 | 6.2 | 7.3 | 10.3 | 8.4 | 9.2 | 8.5 | 5.2 | 3.1 | 426 | 344 |
| 30 | Mixed | Male intact | 11 | 17.3 | AST | Malignant | T2N0M0 | II | 0.1 | 0.2 | 14.3 | 9.6 | 9.4 | 10.3 | 6.7 | 6.8 | 7.2 | 6.1 | 372 | 421 |

[1] Cutaneous mast cell tumors are classified as low-grade or high-grade based on morphological features and proliferative activity [1]; [2] The clinical stage of mast cell tumors is according to the rules based on clinical and histological features [2].

**Table S3.** Distribution of 30 dogs with tumors based on thromboelastography G determined coagulability following tumorectomy.

| TEG G (x $10^3$ dyn/$cm^2$) | Before treatment | After treatment |
|---|---|---|
| Hyper (> 7.2) | 25 (83.3 %) | 24 (80.0 %) |
| Normal (3.2–7.2) | 5 (16.7 %) | 6 (20.0 %) |
| Hypo (< 3.2) | 0 (0.0 %) | 0 (0.0 %) |
| Total (*n*) | 30 (100 %) | 30 (100 %) |

## Reference


1. Kiupel, M.; Webster, J.D.; Bailey, K.L.; Best, S.; DeLay, J.; Detrisac, C.J.; Fitzgerald, S.D.; Gamble, D.; Ginn, P.E.; Goldschmidt, M.H.; et al. Proposal of a 2-tier histologic grading system for canine cutaneous mast cell tumors to more accurately predict biological behavior. *Vet Pathol* **2011**, *48*, 147–155. https://doi.org/10.1177/0300985810386469.
2. Horta, R.S.; Lavalle, G.E.; Monteiro, L.N.; Souza, M.C.C.; Cassali, G.D.; Araújo, R.B. Assessment of Canine Mast Cell Tumor Mortality Risk Based on Clinical, Histologic, Immunohistochemical, and Molecular Features. *Vet Pathol* **2018**, *55*, 212–223. https://doi.org/10.1177/0300985817747325.
